# Do submucous myoma characteristics affect fertility and menstrual outcomes in patients underwent hysteroscopic myomectomy?

**Published:** 2015-06

**Authors:** Ahmed Namazov, Resul Karakus, Ezgi Gencer, Hamdullah Sozen, Levent Acar

**Affiliations:** *Department of Obstetrics and Gynecology, Zeynep Kamil Hospital, Istanbul, Turkey.*

**Keywords:** *submucousal myoma*, *Hysteroscopy*, *Menorrhagia*, *Infertility*

## Abstract

**Background:**

Submucous myomas may be associated with menorrhagia, infertility and dysmenorrhea.

**Objective:**

The aim of this study was to determine the long term effects of submucousal myoma resection on menorrhagia and infertility; also to detect whether the type, size, and location of myoma affect the surgical success.

**Materials and Methods:**

Totally 98 women referred to hysteroscopy for symptomatic submucousal fibroids (menorrhagia (n=51) and infertility (n=47)) between 2005- 2010 were enrolled in this historical cohort study Pregnancy rates and menstrual improvement rates were compared according to myoma characteristics (size, type and location).

**Results:**

After a mean postoperative period of 23±10 months in 51 patients with excessive bleeding, 13 had recurrent menorrhagia (25%). In Other 38 patients excessive bleeding was improved (75%). The improvement rates by location and myoma type: lower segment 100%, fundus 92%, and corpus 63%; type 0) 70%, type 1) 78%, type 2) 80%. The mean sizes of myoma in recurred and improved patients were 23.33 mm and 29.88 mm respectively. 28 of 47 infertile women spontaneously experienced thirty pregnancies (60%). Pregnancy rates according to myoma location and type: lower segment 50%, fundus 57%, and corpus 80%; type 0) 75%, type 1) 62%, type 2) 50%. The mean myoma size in patients who became pregnant was 30.38 mm; in patients who did not conceive was 29.95 mm.

**Conclusion:**

The myoma characetesitics do not affect improvement rates after hysteroscopic myomectomy in patients with unexplained infertility or excessive uterine bleeding.

## Introduction

Leiomyomas are benign smooth muscle tumors that typically originate from the myometrium. Their incidence among women is generally cited as 20-25%, but has been shown to be as high as 70-80% in studies using histologic or ultrasonographic examination (1-2). Prior to fibroid resection an evaluation of the uterine cavity by ultrasonography and diagnostic hysteroscopy permit an accurate classification of fibroids with regard to size, number and locations, which allows selection for trans cervical resection in appropriate cases. Although submucosal fibroids are less frequently encountered, they are more likely symptomatic. They may be associated with menorrhagia, infertility and dysmenorrhea. Bleeding is the most common symptom and usually present as menorrhagia (3). 

There is stronger association of subfertility with submucous leiomyomas than with tumors located elsewhere (4). Most publications addressing submucous myomas report a high pregnancy wastage rate, frequently exceeding 70%. However, the role of the submucous myoma in this regard and indications for myomectomy remain controversial (5). However, indirect evidence that the pregnancy rate in women with otherwise unexplained primary infertility is fairly good, and more than half of patients become pregnant after hysteroscopy surgery (6). Operative hysteroscopy is standard surgical approach and accepted treatment for submucous myomas. We retrospectively analyzed data from a large series of hysteroscopy myomectomies done on 5 years in Zeynep Kamil Training and Research Hospital, First Gynecology Clinic.

The aim of this study was to assess the long term effects of submucous myomectomy on menorrhagia and infertility; also to detect whether the type, size, and location of myoma affect the surgical outcome.

## Materials and methods

A historical cohort study was conducted of women referred to a tertiary care training center specializing in operative hysteroscopy for symptomatic submucousal fibroids (menorrhagia and infertility) between 2005- 2010. The study was approved by the local institutional review board of Zeynep Kamil Hospital. The following data were collected retrospectively: demographics; pregnancy rates before surgery; indications for surgery; duration of infertility; menstrual bleeding pattern; causes of infertility; complications related to procedure. The endometrial cavity was divided into the following three area categories: fundus, corpus and lower uterine segment. Myoma type, size and number were collected based on operation notes. Menorrhagia was diagnosed based on patient history and serum hemoglobin and hematocrit values.

Woman who had been attempting to conceive for at least 12 months was defined as infertile (primary or secondary). Women with persistent anovulation, bilateral tubal occlusion and severe partner dyspermia were excluded from the study. To avoid affecting the results, patients who had in-vitro fertilization in postoperative period were excluded from the study. Patients with multiple myomas were excluded from the study, so all patients had only one submucous myoma. Totally 98 women suffering from infertility and menorrhagia were enrolled to the study, 47 and 51 respectively.

Prior to surgery submucous myoma was diagnosed based on diagnostic hysteroscopy and ultrasonography. Submucous myomas were classified according to the European Society of Gynecologic Endoscopic Classification: type 0 (pedunculated submucous myoma without intramural extension), type I (sessile and with an intramural part of less than 50%) and type II (with an intramural part of 50% or more). Operative hysteroscopy was performed under general anesthesia. 

All patients had prophylactic antibiotics. After dilatation of cervix to Hegar dilator 9 mm, a 26-Fr resect scope was used for resection. Uterus was distended with 1.5% glycine or saline solution according to the type of resecting tool used, with a maximal pressure of 100 mmHg. The inflow and outflow was carefully noted to detect the deficit. The operation time did not exceed 50 min. All specimens were sent for histologic examination. The myoma size was lower than 35 mm in all patients. No complication was observed at intraoperative and early postoperative period. Hospitalization was 24 hr post-operatively. Fertility outcomes were collected at follow up visit or by telephone. Menstrual outcome were evaluated at follow- up visit based on a pictorial blood assessment chart devised by Higham *et al* (7). A monthly score of 100 or higher was associated with menorrhagia.

**Statistical analysis**

Statistical analysis was performed using SPSS software (Statistical Package for the Social Sciences, version 19.0, SPSS Inc, Chicago, Illinois). Chi-Square test and Mann Whitney U test were used for statistical analysis. P<0.05 was considered to be significant. Pregnancy rates and menstrual improvement rates were compared according to myoma characteristics (size, type and location).

## Results

The mean±SD participant age was 35±5 years. In patients suffering from infertility the mean ± SD of infertility duration was 4±4 years. In patients suffering from menorrhagia the duration of excessive uterine bleeding was 11±3 months. Four patients had submucous myoma with deep intramural extension and it was difficult to perform complete resection in these patients. After 2 months later second resection was performed in these patients. Three months postoperatively after complete resection all patients had a hysteroscopy examination. No disease recurrence was observed on postoperative period. 

After a mean postoperative period of 23±10 months menstrual improvement rate was evaluated. In 51 patients who complicated by excessive bleeding, 13 had recurrent menorrhagia (25%). All patients with recurrent menorrhagia underwent hysterectomy at different hospitals in this period. Other 38 patients were classified as an improved patient group (75%). The improvement rates by location and myoma type were as follows: lower segment 100%, fundus 92%, and corpus 63%; type 0) 70%, type 1) 78%, type 2) 80%. 

**Figure 1:**
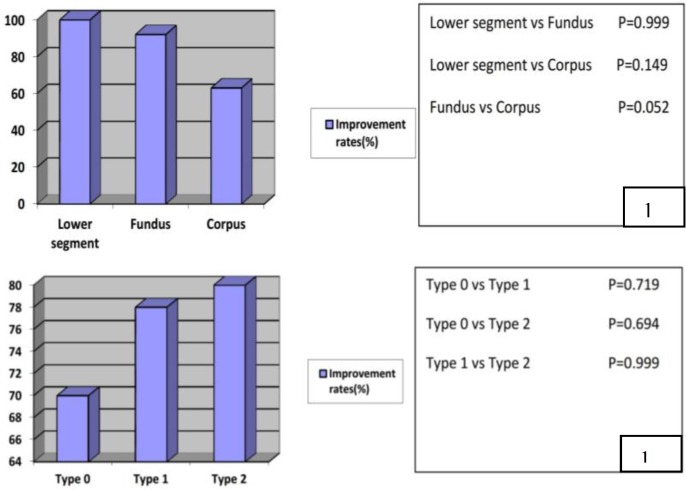
Improvement rates of patients in menorrhagia group according to myoma characteristics (Chi-Square test).

Those variations were not statistically significant (Chi-Square test) (Figure 1). The mean size of myoma in recurred and improved women were 23.33 mm and 29.88 mm respectively (p=0.141, Mann Whitney U test). There was no statistical difference according to the myoma size (Table I). 28 of 47 infertile women spontaneously experienced thirty pregnancies, with an overall 23±10 months postoperatively period (60%). The mean period between myomectomy and conception was 5±1.5 months. 

**Figure 2:**
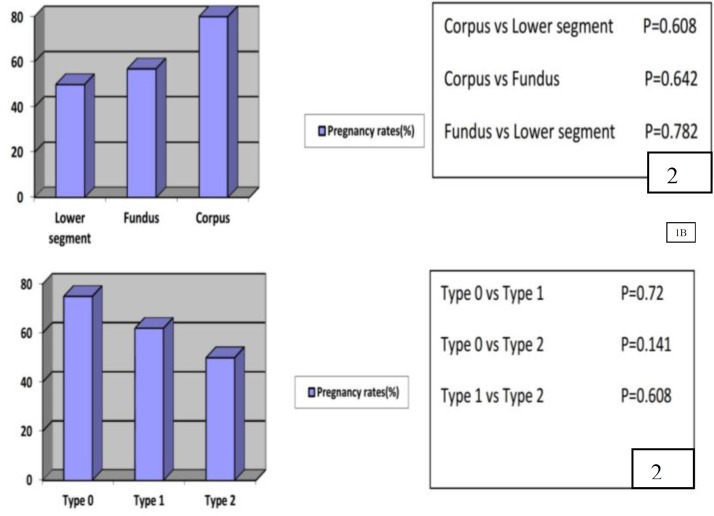
Pregnancy rates in infertility group according to myoma characteristics (Chi-Square test

Pregnancy rates according to myoma location and type are such as following: lower segment 50%, fundus 57%, and corpus 80%; type 0) 75%, type 1) 62%, type 2) %50. Those variations were not statistically significant (Chi-Square test) (Figure 2). The mean myoma size in patients who became pregnant was 30.38 mm, in patients who did not conceive was 29.95 mm and no statistical difference was found (p=0.961, Mann Whitney U test) (Table I).

**Table I:** Mean size of excised myoma in menorrhagia and infertility groups

**Groups**		**Myoma size (mm) **
Menorrhagia group (n=51)		
	Improved	23.30 ± 10
	Recurred	29.88 ± 5
	p-value	0.141
Infertility group (n=47)		
	Conceived	30.38 ± 4
	Not conceived	29.95 ± 5
	p-value	0.961

Data are presented as mean ± SD

Mann Whitney U test

## Discussion

Although submucosal fibroids are less frequently encountered, they are more likely symptomatic. They may be associated with menorrhagia, infertility and dysmenorrhea. There are several pathophysiological explanations for myoma induced menorrhagia. These may include the mechanical compression of veins, the interference with uterine contraction, and the expression of vasoactive growth factors (8-10). Deregulation of vasoactive growth factors is also thought to promote vasodilatation. When engorged venules are disrupted at the time of menstrual sloughing, bleeding from the markedly dilated venules overwhelms usual hemostatic mechanisms (11).

In present study, during the follow-up period bleeding was controlled in 75% of patients with menorrhagia, confirming the satisfactory results obtained by other surgeons (12-14). Hysterectomy was performed in 25% of patients. Loffer reported long-term outcomes of women with hysteroscopy myomectomy with concomitant endometrial ablation and without endometrial ablation (14). Our study comprised patients with menorrhagia who all underwent hysteroscopy myomectomy without endometrial ablation. Loffer reported a hysterectomy rate of 22.1% among women underwent hysteroscopy myomectomy without endometrial ablation (14)

This rate correlates with the hysterectomy rate in the present study (25%). In another retrospective study, among 27 women who had menorrhagia, an improvement occurred in 62% of cases (14). In present study we found that myoma size, location and type did not affect improvement rates in patients with menorrhagia. In a retrospective study, designed by Vercellini *et al* with an overall 3- year cumulative menorrhagia recurrence rate was reported as 30% (15). In present study after a mean postoperative period of 23±10 months the recurrence rate was 25%. Vercellini *et al* reported that recurrence and improvement rates were not significantly influenced by the type of myoma. Our retrospective data support this report. To the best of our knowledge, no data exist regarding menstrual outcome after hysteroscopy myomectomy according to the myoma location and size.

Women with cavity-distorting fibroids who undergo myomectomy are more likely to conceive a pregnancy. In addition, observational studies have reported that the presence of intracavitary leiomyomas decreases pregnancy rates in women undergoing in vitro fertilization. Thus, many IVF units advise women with these lesions to undergo myomectomy. Retrospective and case control studies have demonstrated that submucous myoma resection improves pregnancy rates in patients with infertility as 27-77% (14-18). Our data support these findings: in present study in 23±10 months postoperatively period the rate of spontaneous conception was 60%. 

Our results suggest that pregnancy rates after hysteroscopic myomectomy are not significantly influenced by myoma location, type and size. Tarek Shokeir *et al* in his prospective randomized trial showed no difference in pregnancy rates according to myoma location and size (18). Also Bernard *et al* reported that pregnancy rates were not affected by the myoma location and size (19). In contrast, Varasteh *et al* reported that hysteroscopy polypectomy and myomectomy of myomas larger than 2 cm in diameter improves pregnancy rates (20). We suggest that even myomas smaller than 2 cm in diameter can influence fertility. Because there are many different mechanisms by which myomas may affect fertility. Vercellini *et al* have reported retrospectively pregnancy rates after hysteroscopy myomectomy (16). They found no statistical difference in pregnancy rates according to myoma type. 

However, Tarek Shokeir *et al* have reported: according to the type of the ﬁbroid, the myomectomy procedure was associated with signiﬁcantly higher pregnancy rates in patients with type 0 and type I myomas (18). In the present study, pregnancy rates in type 0 and 1 myomas were found higher than in type 2, but this was not statistically significant. We suggest that, although intracavitary part in type 0 and type 1 myoma is larger than in type 2 myoma, intramural part also influences infertility. Because myomas affect fertility not only by cavity deformation, but increased uterine contractility, endometrial inflamation, impaired endometrial blood flow also influence fertility outcome (5). In the current study the mean period between myomectomy and conception was 5±1.5 months. This short period for conception has also been reported by others (16, 18). It is recommended that patients conceive soon after the period.

According to our study the myoma characetesitics do not affect improvement rates after hysteroscopy myomectomy in patients with unexplained infertility or excessive uterine bleeding. Large prospective randomized trials could be designed, to assess the relationship between submucous myoma characteristics and postoperative outcomes. But we think that in symptomatic patients (menorrhagia and infertility) with submucous myoma, an expectant management will not be ethical. So randomized-controlled trials will be difficult to design.

## References

[1] Cramer SF, Patel A (1990). The frequency of uterine leiomyomas. Am J Clin Pathol.

[2] Baird DD, Dunson DB, Hill MC, Cousins D, Schectman JM (2003). High cumulative incidence of uterine leiomyoma in black and white women: ultrasound evidence. Am J Obstet Gynecol.

[3] Olufowobi O, Sharif K, Papaionnou S, Neelakantan D, Mohammed H, Afnan M (2004). Are the anticipated benefits of myomectomy achieved in women of reproductive age? A 5 year review of the results at a UK tertiary hospital. J Obstet Gynaecol.

[4] John O (2008). Schorge Williams Gynecology.

[5] Taylor E, Gomel V (2008). The uterus and infertility. Fertile Steril.

[6] Pritts EA, Parker WH, Olive DL (2009). Fibroids and infertility: an updated systematic review of the evidence. Fertil Steril.

[7] Higham JM, O’Brien PM, Shaw RW (1990). Assessment of menstrual blood loss using a pictorial chart. Br J Obstet Gynaecol.

[8] Farrer-Brown G, Beilby JO, Tarbit MH (1970). The vascular patterns in myomatous uteri. J Obstet Gynaecol Br Commonw.

[9] Farrer-Brown G, Beilby JO, Tarbit MH (1971). Venous changes in the endometrium of myomatous uteri. Obstet Gynecol.

[10] Stewart EA, Nowak RA (1998). New concepts in the treatment of uterine leiomyomas. Obstet Gynecol.

[11] Stewart EA, Nowak RA (1996). Leiomyoma- related bleeding: a classic hypothesis updated for the molecular era. Hum Reprod Update.

[12] Wamsteker K, Emanuel MH, de Kruif JH (1993). Transcervical hysteroscopic resection of submucous fibroids for abnormal uterine bleeding: Results regarding the degree of intramural extension. Obstet Gynecol.

[13] Fernandez H, Sefrioui O, Virelizier C, Gervaise A, Gomel V, Frydman R (2001). Hysteroscopic resection of submucosal myomas in patients with infertility. Hum Reprod.

[14] Loffer FD (2005). Improving results of hysteroscopic submucosal myomectomy for menorrhagia by concomitant ablation. J Minim Invasive Gynecol.

[15] Vercellini P, Zàina B, Yaylayan L, Pisacreta A, De Giorgi O, Crosignani PG (1999). Hysteroscopic myomectomy: long term effects on menstrual pattern and fertility. Obstet Gynecol.

[16] Goldenberg M, Sivan E, Sharabi Z, Bider D, Rabinovici J, Seidman DS (1995). Outcome of hysteroscopic resection of submucous myoma for infertility. Fertil Steril.

[17] Corson SL, Brooks PG (1991). Resectoscopic myomectomy. Fertil Steril.

[18] Shokeir T, El-Shafei M, Yousef H, Allam AF, Sadek E (2010). Submucous myomas and their implications in the pregnancy rates of patients with otherwise unexplained primary infertility undergoing hysteroscopic myomectomy: a randomized matched control study. Fertil Steril.

[19] Bernard G, Darai E, Poncelet C, beniﬂa JL, Madelenat P (2000). Fertility after hysteroscopic myomectomy: effect of intramural myomas. Eur J Obstet Gynecol Reprod Biol.

[20] Varasteh NN, Neuwirth RS, Levin B, Keltz MD (1999). Pregnancy rates after hysteroscopic polypectomy and myomectomy in infertile women. Obstet Gynecol.

